# Navigating intimate sexual partnerships in an era of HIV: dimensions of couple relationship quality and satisfaction among adults in Eswatini and linkages to HIV risk

**DOI:** 10.1080/17290376.2019.1604254

**Published:** 2019-04-15

**Authors:** Allison Ruark, Edward C. Green, Amy Nunn, Caitlin Kennedy, Alfred Adams, Thandeka Dlamini-Simelane, Pamela Surkan

**Affiliations:** a Department of Medicine, Brown University, Providence, RI, USA; b Anthropology, George Washington University, Washington, DC, USA; c Brown University School of Public Health, Providence, RI, USA; d Rhode Island Public Health Institute, Providence, RI, USA; e Johns Hopkins University Bloomberg School of Public Health, International Health, Baltimore, MD, USA; f University of Amsterdam, Amsterdam Institute for Social Science Research, Amsterdam, Netherlands

**Keywords:** Multiple sexual partnerships, concurrency, sexual satisfaction, love, respect, couple communication

## Abstract

Couple relationship functioning impacts individual health and well-being, including HIV risk, but scant research has focused on emic understandings of relationship quality in African populations. We explored relationship quality and satisfaction in Eswatini (formerly Swaziland) using data from 148 in-depth interviews (117 life-course interviews with 28 adults and 31 interviews with 29 marriage counselors and their clients) and 4 focus group discussions. Love, respect, honesty, trust, communication, sexual satisfaction, and sexual faithfulness emerged as the most salient characteristics of good relationships, with both men and women emphasising love and respect as being most important. Participants desired relationships characterised by such qualities but reported relationship threats in the areas of trust, honesty, and sexual faithfulness. The dimensions of relationship quality identified by this study are consistent with research from other contexts, suggesting cross-cultural similarities in conceptions of a good relationship. Some relationship constructs, particularly respect, may be more salient in a Swazi context.

## Introduction

A wealth of data from Western contexts demonstrates the linkages between intimate sexual partnerships and personal well-being, although far less is found in the literature regarding couple relationships in other cultural settings (Mastekaasa, 1994). Marital quality has consistently been shown to influence individual health and well-being (Proulx, Helms, & Buehler, 2007) and marital satisfaction has been associated with happiness in life (Carr, Freedman, Cornman, & Schwarz, 2014).^1^ Marriage may provide ‘protection’ when individuals encounter stressful life events and health problems, and married individuals may be more likely to practice healthy behaviours (Ren, 1997). In contrast, marriages characterised by conflict and low satisfaction are correlated with poor health (Ren, 1997; Whitson & El-Sheikh, 2003). Most couple relationship research in the United States and other Western contexts has been conducted with married couples, and the generalisability of this research to unmarried couples or non-Western contexts warrants further study. For example, research has been inconclusive regarding whether the health benefits of marriage extend to cohabiting couples (Brown, Bulanda, & Lee, 2005; Drefahl, 2012; Koskinen, Joutsenniemi, Martelin, & Martikainen, 2007; Marcussen, 2005; Perelli-Harris & Styrc, 2018; Ren, 1997; Wiik, Keizer, & Lappegård, 2012).

To date, most research of couples in Africa has focused on violence and power, with less attention to more positive aspects of couple relationships such as love and intimacy (Ruark, Stern, Dlamini-Simelane, & Kakuze, 2017). Gender-based violence and other forms of gendered power inequity are well recognised as having serious detrimental effects on multiple aspects of women’s health (Blanc, 2001; Hatcher et al., 2012; Siedner et al., 2012), including risk of HIV (Dunkle et al., 2004; Jewkes, Dunkle, Nduna, & Shai, 2010). Conversely, shared power between partners has been linked to higher relationship quality (Conroy, McGrath, et al., 2016).

Researchers have also explored relationship quality among African couples in the context of HIV prevention (Belus, Kline, Carney, Myers, & Wechsberg, 2018), uptake and experience of HIV testing and counselling (Conroy, 2014, 2015; Darbes et al., 2014; Maman, Mbwambo, Hogan, Kilonzo, & Sweat, 2001; Tabana et al., 2013), and adherence to HIV treatment (Conroy, McKenna, & Ruark, 2019; Conroy et al., 2017). A qualitative study with young South African couples found that relationships characterised by mistrust, poor communication, and violence placed them at greater risk of HIV infection (Parker, Pettifor, Maman, Sibeko, & MacPhail, 2014), and young South African men who reported having positive dating relationships were more likely to report condom use (Gevers, Jewkes, & Mathews, 2013). In Uganda, an innovative qualitative case–control study found that compared to HIV-negative individuals, HIV-positive individuals had poorer-quality relationships characterised by deficient communication and greater suspicion and mistrust, including not being sure if one’s partner had other sexual partners (Higgins et al., 2014).

In addition, epidemiological data provide clear evidence that risk of HIV is closely related to the type of sexual partnership (Bongaarts, 2007). Although previous research had found that marriage was associated with higher HIV prevalence for young women in African contexts with high rates of early marriage (Clark, 2004), more recent evidence from southern Africa (where marriage occurs later and is less normative) has found marriage to be protective. Swazi women who are married and living with their spouses have one-third the HIV incidence of unmarried women (Justman et al., 2017). In neighbouring South Africa, women and men who are married and living with their spouses have one-tenth the HIV incidence of unmarried, cohabiting women and men (Shisana et al., 2016).

The current study is part of a larger project addressing how dynamics of sexual partnerships impact HIV risk among Swazi adults (Ruark et al., 2014, 2016). Previous research found a high level of risky sexual behaviours, particularly concurrent sexual partnerships, and that women and men sought multiple partners in part because they were sexually and emotionally dissatisfied in their relationships (Ruark et al., 2014). Although participants reported desiring high-quality and satisfying relationships, most felt their relationships fell far short of these ideals, and problems in their primary relationships led to behaviours which placed them at high risk of HIV infection (Ruark et al., 2016).

Given these realities and the observed linkages between relationship quality and HIV risk, the primary goal of the current study is to more fully describe how young Swazi adults answer the question, *What is a good relationship?* We qualitatively investigate the characteristics of a good relationship (relationship quality) and to what degree participants felt these characteristics were or were not present in their own intimate sexual partnerships (relationship satisfaction). We posit that understanding how Swazis conceptualise their own relationships is critical to deciphering the linkages between relationship dynamics and health outcomes, particularly HIV risk. A secondary goal of the study was to explore the potential of a culturally-grounded response to support couples in building healthier relationships.

## Methods

### Study setting

The current study was carried out in Eswatini (formerly Swaziland), a small country in southern Africa with an estimated population of 1.4 million people. Nearly one in three adults in Eswatini is HIV-infected, with HIV prevalence of 39% among women and 24% among men (Bicego et al., 2013), and annual HIV incidence of 3.1% among women and 1.7% among men (Justman et al., 2017). HIV is primarily spread through heterosexual sex (National Emergency Response Council on HIV and AIDS (NERCHA), 2009). In a recent seroincidence survey, having a partner of unknown sero-status was significantly associated with higher HIV incidence for women and men, and for women, being unmarried or being married but living apart from one’s spouse were also associated with greater HIV incidence (Justman et al., 2017).

Rates of marriage in Eswatini have declined in recent decades (Ndlangamandla, 2007) and according to the most recent Demographic and Health Survey less than half of adults were married or cohabiting (Central Statistics Office (CSO) & Macro International Inc., 2008). Among women aged 20–49 years, 42% were married and 12% were living with a partner, and among men aged 20–49 years, 34% were married and 9% were living with a partner.^2^ Polygamy was relatively rare, with only 4% of urban men and 7% of rural men reporting having more than one wife (Central Statistics Office (CSO) & Macro International Inc., 2008). The great majority of the population of Eswatini is ethnic Swazi, as were all participants in this research. Nearly 9 in 10 Swazis identify with various Christian sects, and approximately three-quarters of Swazis live in rural areas (Central Statistics Office (CSO) & Macro International Inc., 2008).

### Data collection

We collected data in three phases, with three different groups of participants: life-course interviews with Swazi women and men in their 20s and 30s, focus group discussions (FGDs) with Swazi women and men in their 20s and 30s, and in-depth interviews with marriage counselors and their clients (counselees) who were over the age of 20 (but with no upper age limit). The life-course interviews aimed to capture data on sexual partnerships as well as investigate transitions and trajectories of these partnerships, using a life-course approach. A life-course approach analyses the events of a person’s life, as well as the socially-constructed roles he or she enacts, over time (Giele & Elder, 1998). Such an approach allows for examination of how sociocultural and structural factors shape the individual, as well as how past experiences shape present experiences. The FGDs gave participants an opportunity to engage with each other on topics such as social norms and gender roles in sexual relationships. Marriage counselors served as key informants regarding couple relationships in Eswatini, and interviews with counselees allowed us to investigate relationship challenges in more depth and explore the potential for marriage counselling to strengthen couple relationships in the Swazi context. Data collection took place between July 2013 and August 2014.

### In-depth interviews and focus group discussions

We conducted 117 repeated, in-depth, life-course interviews: 59 interviews with 14 women and 59 interviews with 14 men. Given the goal of sampling participants who were characteristic of young Swazi adults, we recruited participants in shopping centres or outdoor spaces in central Mbabane, the capital of Eswatini, using central intercept sampling (Butler, 2008). Eligibility criteria were being ages 20–39 and ever having had sex. We focused the study on young adults in their 20s and 30s as this is the cohort at highest risk of HIV infection. We recruited participants in two rounds, reviewing participant data between rounds in terms of participants’ age, socioeconomic status, education level, and marital status. In the second round of recruitment, we purposively sampled participants to achieve maximum variation, specifically recruiting participants who were in their 30s and married.

Each participant was interviewed at least three times (maximum five times) over a period of 1–13 months (average 9 months) using a life-course approach, which focused on life experiences from childhood to the present. Interviews were conducted by same-gender Swazi researchers who had been trained in qualitative research methodologies, in siSwati or in a mixture of English and siSwati, and took place at the participant’s home or in a private office. Each participant was interviewed until all topics in the interview guide had been explored and saturation had been reached, for a total of two to four hours of interviews per participant. Interviews were semi-structured and iterative, allowing for exploration of themes in subsequent interviews based on emerging findings or insights from earlier interviews. Our data collection process was also iterative, allowing us to refine the interview guide over the course of data collection.

In addition, 19 women participated in two FGDs (*n_1 _*= 13, *n_2 _*= 6), and 13 men participated in two FGDs (*n_1 _*= 7, *n_2 _*= 6). We recruited participants ages 20–39 in shopping centres or outdoor spaces in central Mbabane using central intercept sampling (Butler, 2008) and invited them to participate in an FGD on that day or the subsequent day. FGDs were held in a private office. Same-gender Swazi researchers (AKA and TD) conducted the FGDs in siSwati. Each FGD lasted between an hour and a half and two hours.

Interviews and FGDs were carried out using semi-structured interview guides including the following questions: *What things make a good relationship? How should a man treat a woman? How should a woman treat a man? What things make you* [*or a man or woman*] *want to stay with a partner?* For life-course interviews, discussions of relationship satisfaction and quality were situated within a discussion of sexual partnerships over participants’ lifetimes. Participants were asked to describe each sexual partnership in as much detail as possible, including how the partnership had started and ended and their motivations and expectations for that partnership. These life-course data have been further described elsewhere (Ruark et al., 2014, 2016).

### Ranking of relationship characteristics

Using data from the first two FGDs (one with men, one with women), we generated a list of 15 relationship characteristics for use in a ranking exercise. Life-course interview participants ranked these relationship characteristics (from 1 to 15) by their importance to an intimate sexual relationship. We generated for each characteristic a measure of salience equal to that item’s average rank across participants, using ANTHROPAC 4.98 (Analytic Technologies, Lexington, Kentucky, USA).

### Interviews with marriage counselors and counselees

In addition, we conducted 19 in-depth interviews with 17 marriage counselors (8 women and 9 men, including 2 married couples), and 12 interviews with 12 married counselees (6 women and 6 men, including 4 married couples). We recruited counselors and counselees through referrals from counselor training programmes, and sampled with the goal of achieving diversity in terms of age, gender, and occupation. All counselors were lay (non-professional) counselors who provided counselling for little or no compensation to couples in their churches or social networks who requested counselling before marriage or at other times. All counselors were Christians and had been trained through church-affiliated programmes, and 7 of the 17 counselors were pastors.

Interviews were carried out in siSwati by Swazi researchers (21 interviews), or in English by the first author (10 interviews), according to the language preference of the participant. Couples were interviewed separately. Interviews were held in private locations using a semi-structured interview guide, and lasted from half an hour to two hours. Counselors were asked to describe their training and work, including specific examples of couples they had counselled. Counselees were asked to describe their experiences being counselled, including what relationship challenges had caused them to seek counselling and perceived benefits of counselling.

### Analysis

All interviews and FGDs were audio recorded, transcribed verbatim, and translated from siSwati into English, if necessary. Interviewers wrote memos describing their observations immediately after each interview and promptly transcribed and translated the interview. The first author reviewed and gave detailed feedback to each interview, including guidance on what topics to explore in the next interview, which ensured data quality and promoted ongoing, collaborative analysis amongst the study team. In addition, the study team (including Swazi researchers) held frequent meetings to identify themes and discuss interpretation of data. We also attempted to increase the credibility and trustworthiness (Lincoln & Guba, 1985) of the research through member checking with research participants and discussion of findings with other Swazis knowledgeable about these topics, triangulation between data sources (such as in-depth interviews and FGDs), and close attention to negative cases and how participants chose to represent themselves in the research.

Given the lack of previous research and theory regarding relationship quality and satisfaction among Swazis or similar populations, we used conventional content analysis as described by Hsieh and Shannon (2005) and approached the data with as few presuppositions and assumptions as possible. The first author coded data, including transcripts and memos, with open and axial codes (Creswell, 2013) derived from the data themselves, and using NVivo 10 (QSR International, Doncaster, Victoria, Australia). Characteristics of good relationships (named with commonly used siSwati terms), as well as statements about relationship satisfaction, were treated as core concepts during open coding, and constant comparisons were made between how different participants discussed these concepts. Axial coding was used to investigate questions of how participants understood, experienced, and described relationship satisfaction; how these understandings were socially constructed; the characteristics of good relationships; and differences between subpopulations, particularly men and women. Content analysis (Brenner, 1985) was utilised to assess how often participants in the life-course interviews mentioned various relationship characteristics.

### Ethics

The study was approved by the Institutional Review Board of The Miriam Hospital (Providence, Rhode Island) and the Scientific and Ethics Committee of the Ministry of Health in Eswatini. All participants provided written informed consent, with the exception of FGD participants, who provided verbal consent. In addition, all participants were offered cell phone vouchers worth 20 Swazi Emalangeni (approximately 2 USD) for participation in each interview or FGD.

## Findings

Participants in this study were diverse in terms of education level, profession, and rural or urban residence. Only 6 of 28 life-course interview participants were married at the start of research, while 6 were cohabiting with a partner, 15 were in a relationship but not cohabiting, and 1 was not in a relationship. Of 32 FGD participants, 7 were married. Participants in the life-course interviews will be described throughout this article as either married, cohabiting, single, or partnered (in a relationship but not cohabiting). All of the marriage counselors had been married, but 4 were widowed or separated at the time of research, and they had a mean age of 55. Counselees were primarily in their 30s, with a mean age of 39, and all were married. As most quotes in this article are from life-course interview participants, type of interview is indicated only for quotes from FGD participants, counselors, and counselees.

In the ranking exercise, life-course interview participants ranked love as the most important relationship characteristic. Men ranked love higher (average rank 1.7) than did women (average rank 4.0) (Table 1). Love, respect, honesty, trust, and communication were the top-ranked relationship characteristics for both men and women, and for both married and unmarried individuals. Most other characteristics were ranked similarly among all sub-groups.

**Table 1.** Average rank of relationship characteristics among life-course interview participants.

	Avg. rank (N = 28)	Women (N = 14)	Men (N = 14)	Married (N = 6)	Unmarried (N = 22)
love	2.9	4.0	1.7	5.0	2.3
respect	4.0	3.9	4.0	4.5	3.8
honesty	4.1	4.4	3.8	5.2	3.8
trust	5.1	6.0	4.2	5.3	5.1
good communication	6.1	6.9	5.4	7.0	5.9
shared spiritual life	8.1	6.3	10.0	5.7	8.8
conflict resolution	8.4	8.4	8.3	6.0	9.0
saying ‘I love you’	8.9	8.3	9.6	8.8	9.0
sexual satisfaction	9.0	10.1	7.9	9.2	8.9
sexual faithfulness	9.1	9.7	8.4	8.7	9.2
spending time together	9.3	8.4	10.2	11.0	8.8
financial support	9.3	8.2	10.4	9.8	9.2
physical attraction	11.3	12.2	10.3	12.0	11.1
practical service	12.0	11.3	12.7	11.3	12.2
physical affection	12.5	11.9	13.1	10.5	13.1

Note: Items are ranked on a scale of 1 (most important) to 15 (least important).

When asked to describe what makes a good relationship, participants in the life-course interviews most often mentioned the characteristics of love, respect, honesty, trust, communication, sexual satisfaction, and sexual faithfulness. Five of these characteristics were also ranked as most important in the ranking exercise. The remaining two characteristics, sexual faithfulness and satisfaction, received significant attention across all forms of data collection. Table 2 shows how many times participants in the life-course interviews mentioned these seven characteristics as being important or present in their relationships. Love and respect were most frequently mentioned. In response to the question, *What makes a good relationship?*, love was most frequently mentioned. Respect was most frequently mentioned in response to questions about how women and men should treat each other within relationships.

**Table 2.** Frequency of mention of relationship characteristics during life-course interviews.

	Women (N = 14)	Men (N = 14)
love	102	90
respect	94	74
sexual faithfulness	46	65
good communication	48	45
honesty	30	32
trust	27	31
sexual satisfaction	20	15

When asked how a woman should treat a man, most female participants in the life-course interviews (11 of 14) first discussed respect, submission, or doing what a male partner wanted (using phrases such as, ‘she has to do everything she is told to do by him’). Women also were most likely to mention respect first when asked how a man should treat a woman. In contrast, a third (5 of 14) of male participants in the life-course interviews first mentioned love when asked how a woman should treat a man, and a majority of men first mentioned love when asked how men should treat women. In sum, women emphasised respect and men emphasised love when discussing how men and women should treat each other.

### Love

Love (*lutsandvo*) was the most discussed characteristic of intimate relationships in the life-course interviews, and was also frequently discussed in the FGDs and interviews with counselors and counselees. Participants described love as foundational and essential to a relationship, as something they needed, and as the origin of several other relationship characteristics including honesty, trust, respect, sexual fidelity, and good communication. A man in his 20s (partnered) remarked, ‘first is love, then love will bear all other fruits … faithfulness, caring for your loved one, trust, and most importantly respect.’

In contrast, the accounts of some women in long-term relationships were notable for not discussing love between themselves and their partners, and for occasional cynicism regarding love. One woman (30s, married) reflected,

I used to have it [love] and now I do not know how to explain it because I don’t have it … Is there such a thing as love? You know you will think that people they love each other … but then the next thing you will see the husband with another lady.

Women who did not feel they had experienced love in their relationships were clear about their desire for it, as the woman in her 20s and not in a serious relationship who said, ‘I wish to get a person who will love me … He must show that he truly loves me, because there are many ways to show a lady that you truly love her besides having sex with her.’ Women also felt that not every man who professed love could be trusted, and one woman (cohabiting, 20s) expressed a common sentiment when she said, ‘[Love] is a word most males use to get in bed with you, and I’m talking from my experience.’

Women and men repeatedly stated that love should be shown through actions. Women emphasised in the life-course interviews and FGDs that men should show love through gifts, financial support, providing for children, sexual fidelity, verbal affirmation of their love, and not keeping secrets. One woman (20s, married) related, ‘I can say that [my husband] loves me and I can see that … because he provides me with everything I need.’ Men emphasised the importance of women showing love through caring for them in practical ways such as cooking and cleaning.

Men and women agreed that being honest and not having additional sexual partners was a sign of love, as was taking steps towards marriage such as formal introductions to the partner’s family. One woman in her 30s reported that her boyfriend had stopped having other sexual partners, concluding, ‘Now I know that [he] loves me.’ A married man in his 30s remarked:

If I say a man should love his wife I mean that a man should show his wife that he loves her … women are quick to see that they are still loved … I can say [love] is the most important characteristic out of all the others. I love my wife very much. That is why I have to be honest [and] I have to be faithful.

### Respect

Love and respect (*inhlonipho*) were often mentioned in tandem, and a number of counselees and participants in the life-course interviews described having relationships of mutual love and respect. Some participants asserted that respect was the most important aspect of a relationship, such as the man (20s, partnered) who said, ‘Respect is our Swazi way of life … respect is the key to every relationship.’ Participants valued the way that their partners showed them respect by including them in decision making, learning about and honouring their desires and preferences, and caring for their needs. A cohabiting man in his 30s remarked, ‘I respect [my partner] in every way …  Now that we are old, we are able to communicate everything with respect. When she has a problem she comes to me and we discuss everything as a family.’

Women and men discussed respect in strongly gendered ways. Respect was often defined (by both genders) as women submitting to men’s wishes, sometimes using phrases such as that the man is the woman’s ‘earthly king.’ Women were held to more restrictive standards than men on matters ranging from household labour to sexual conduct, and this double standard was defended by men and at times women on the grounds that women needed to show respect. Respect was sometimes invoked to justify a code of silence around male infidelity and other relationship problems. Men seemed to have a very low tolerance for disrespectful behaviour, whereas it was evident that some women tolerated what they considered to be a serious lack of respect in their relationships. One man (30s, cohabiting) commented:

I think that there is no man that can want to stay with a partner that does not respect him. Men who are not happy in their marriages or relationships are the ones that are not respected by their women.

Some men and women also argued for the importance of men showing women respect by not engaging in behaviours such as staying out late at night drinking alcohol and having other sexual partners. In one FGD, a man in his 30s expressed, ‘There is need for respecting each other but that respect should not be one-sided, meaning that you as a husband should not expect your wife to respect you, yet you do not respect her as your wife.’ Another man agreed, ‘It is important that we respect our wives because if we do they will give us the love back.’

Like love, respect was also strongly linked to the concept of sexual faithfulness, with a several participants stating that being faithful was proof of respect or that respect was lacking when someone was unfaithful in a relationship. One man (20s, partnered) explained: ‘Everything you do in a relationship should show that you respect each other. If you decide to cheat, it means you do not respect your partner.’ In fact, this man was one of several who had multiple sexual partners while verbally affirming the importance of faithfulness.

### Honesty and trust

Participants described highly valuing honesty (*kwetsembeka*) and trust (*kwetsembana*), although many also admitted that their relationships were characterised by a high degree of secrecy and distrust. Whereas participants typically expressed confidence in the fact that their partners loved them, they were often less sure if their partners were being completely honest or trustworthy. One man (20s, partnered) stated, ‘Some people out there say it is simple to love but difficult to trust a person.’ Trust and honesty were described somewhat interchangeably as sharing money, not keeping secrets, and not having secret sexual partners. Honesty was called the same as being faithful, and a means of ‘protecting your family’ from the threat of HIV. Trust was variously seen as being crucial to love (‘love without trust is incomplete’) and as being an impossible expectation (‘a human being cannot be trusted’).

Letting one’s partner know one’s whereabouts was discussed as a particularly important form of trust. Participants in this study often lived apart from their sexual partners, which created opportunities for secret sexual partnerships. One female FGD participant described the lack of trust in her relationship by saying, ‘Even now we still don’t trust each other. He doesn’t know that I’m in Mbabane and I don’t know where he is.’ In contrast, sharing information and giving one’s partner free access to one’s home signified that one had nothing to hide.

I gave him my love and respect and I am honest with him and he knows that. I told him that he can come at any time at my place and I make sure that I tell him my whereabouts when I need to go somewhere. (woman, 30s, partnered)

Participants also repeatedly mentioned that giving one’s partner access to one’s cell phone was a sign of trust, as the woman (30s, partnered) who said this about her partner:

He has changed, because he can’t allow me to answer his phone knowing very well that he is hiding or doing something he doesn’t want me to know about. So I think that this [giving access to his phone] is his way of trying to show me that he is now faithful.

In life-course interviews, women often reported loving partners they admitted they did not trust completely, whereas men were more likely to say that if a relationship did not have honesty and trust, it could not last. One woman (20s, partnered) claimed that her partner ‘told [her] all of his secrets,’ then later in the same interview stated that she would ‘give him 95% for the trust and the 5% I just don’t trust him.’ She discovered weeks later that he had a ‘secret lover.’ Some female participants in the life-course interviews described a kind of reciprocal distrust in which they decided that their partner’s lack of honesty justified their own lack of honesty, or that a partner’s sexual infidelity was grounds for their decision to engage in a concurrent sexual partnership. One woman with concurrent sexual partners (20s, partnered) admitted in a life-course interview:

Sometimes I’m not honest because I also don’t know what [my partner] is busy with. It’s difficult to trust because a person can change. It takes some time to deeply trust a person. Every person has secrets. I can come and say, ‘This person is like this,’ yet I do not know. But it is important to trust each other and be honest.

In contrast, some participants expressed that they had mutual trust in their relationships, as the woman in an FGD who described a relationship by saying, ‘I trusted him and he trusted me and even before the trust he was my friend.’ Counselees were particularly likely to describe having relationships of mutual trust, perhaps due to the fact that getting married and seeking marriage counselling were steps that required and likely increased trust.

### Communication

Participants in the life-course interviews discussed the many functions that communication served in their relationships, including informing each other of personal likes and dislikes (enabling them to respect each others’ preferences), sharing opinions, making sexual desires known, resolving conflicts and apologising after wrongdoing, communicating love and appreciation, and making decisions and plans together. Several women mentioned communication as being the most important part of a relationship. Women were particularly likely to mention the importance of having their partners listen to them and provide advice and emotional support during difficult times, although several men said that their wives or girlfriends were trusted partners in making decisions or solving life problems. One man (30s, cohabiting) voiced his satisfaction by saying, ‘I have a partner who I share my problems with, she gives me good advice, and there is a person that tells me “I love you” every day.’

Men and women were both concerned with women communicating respectfully, and several men and women said that if women communicated disrespectfully, such as by shouting, this might cause a man to leave the relationship or be unfaithful. A woman explained in an FGD that ‘too much talking drives a man away’ while a man (30s, cohabiting) stated that ‘if [a woman] shouts the relationship will never last because the man will not tolerate such behaviour.’ One woman recounted in an FGD the importance of women communicating respectfully when their partners were angry or cross, rather than starting an argument or jumping to conclusions such as that the partner was having an affair. Another woman in the FGD agreed that it was a woman’s job to relieve her partner’s stress, and rather than waiting for him to relate to her lovingly after a hard day she should tell him, ‘I love you, my love for you is as big as a bus!’ Men in one FGD also discussed the importance of talking to their partners gently during a conflict, and not shouting.

Some participants did not report experiencing conflict in their relationships, and others seemed to be concerned with avoiding or minimising conflict, using statements such as ‘where there is good communication there will be no conflict.’ However, other participants reported serious communication difficulties, and several women reported patterns of physical abuse and violence during conflicts, suggesting that very little positive communication was occurring during conflict. Some participants stated that the main challenge they faced in their relationships was lack of communication. One woman (30s, partnered) was particularly insistent about the value of communication. She blamed her partner’s past infidelities on a lack of good communication in their relationship, which changed after her sister intervened to encourage them to communicate more openly.

He left me for another person because we didn’t communicate … We had problems but we never talked about it, even if he did something bad … Now I see the difference, he is no longer the same as before … The best thing in our relationship is that now we are able to communicate … and he says, ‘I almost lost something precious, a good wife.’

Problems with communication undergirded many other relationship difficulties, including lack of sexual satisfaction, lack of financial support, and infidelity. A cohabiting man (30s) expressed, ‘Sexual satisfaction is important because if you do not satisfy your partner, what do you expect her to do? It is important that you talk about such issues with your partner.’ Some women also discussed the importance of communication about sex. One woman (30s, married) said that if a woman was sexually unsatisfied she should tell her partner to ‘do it again,’ while a female counselee (30s) stated that a woman should not wait for her husband to ask her to have sex, but should ‘be free and tell him that [she] wants to make love.’

Numerous participants discussed the stress and distrust that arose when a couple did not share information about finances, with a woman often assuming when money was tight that her partner was actually supporting a secret lover. Open communication, including disclosing incomes and budgeting together, eased these tensions and caused women to be understanding during financial hardship as they no longer feared that household resources were being diverted to an ulterior use. Many participants expressed a desire for help in learning how to better communicate, and several participants reported having sought advice from family members, counselors, or friends. One woman (30s) who had received marriage counselling spoke of the way that counselling had helped her learn to communicate, saying, ‘I am reserved. But the counselor helped me realise that there was nothing like that in marriage. We have to communicate, in every way.’

### Sexual faithfulness and sexual satisfaction

Sexual faithfulness (*kwetsembeka kutelicansi*) and sexual satisfaction (*kwenetiseka ngelicansi*) were mentioned prominently in all interviews and FGDs. The characteristics of love, respect, honesty, trust, and communication were all frequently discussed in relationship to sexual faithfulness. In fact, the siSwati phrase for sexual faithfulness contains the word ‘honesty.’ The issue of sexual satisfaction was in turn seen as being indivisible from that of faithfulness, with lack of sexual satisfaction being invoked as the main cause of sexual infidelity (often referred to as ‘cheating’) for both genders in all the FGDs and by most of the interview participants. A female participant (30s, single) said, ‘You have to satisfy each other sexually so that your love can flourish and that there will be no cheating.’ A male participant (20s, partnered) stated, ‘There is no relationship that can survive without having sex and satisfying each other in bed.’

The great majority of participants in all phases of this study, male and female, expressed a desire for partners who were sexually faithful, and many also acknowledged the importance of being faithful if one wanted to avoid sexually transmitted infections such as HIV. A majority of participants in the life-course interviews had both engaged in sexual infidelity and had a partner be unfaithful, and these infidelities were the source of considerable emotional pain, particularly for those who had been cheated on. Male participants in the life-course interviews and FGDs repeatedly emphasised the importance of women being sexually appealing and faithful. Several men also mentioned that they realised they might lose partners they loved if they were not faithful, or if their partner discovered their infidelity,

Both men’s and women’s FGDs were notable for their sexually explicit conversation, and sexual satisfaction was one of the major topics – if not the major topic – of conversation. In one FGD, women discussed sexual satisfaction at length and enumerated the things they could do to be sexually appealing and available to their partners. They universally agreed with the woman who said, ‘[Your partner] will leave you and look for someone with experience. That’s why I say in the bedroom do whatever he wants you to do for his satisfaction.’ Participants in this FGD also expressed doubt that any men were sexually faithful, with one participant saying that 95% of men were ‘rotten’ and another adding that ‘there are no more real husbands.’ In the other women’s FGD, a woman remarked,

What I have noted is that the secret lovers take our husbands because they are clean and they look after themselves. So I have to do all that is in my power and fight for him that so he stays and will not be tempted and end up having extramarital affairs.

In one men’s FGD, men were similarly concerned about women’s lack of sexual fidelity, discussing their fears that their partners would acquire other sexual partners if they did not sexually satisfy them and saying that ‘women out there are hunting for men.’ Another man added that other men (graphically referred to as *lamangce*, birds of prey) might steal away their partners ‘because they will do all the things that you were not able to do for her.’ Men in the other FGD debated whether faithfulness was possible. One man claimed it was ‘impossible’ for a man to not have multiple sexual partners, even if married. Two other men argued that faithfulness was achievable because ‘one mistake can be your death,’ and the head of a household should not bring disease into his family (both references to HIV).

Marriage counselors repeatedly emphasised the importance of sexual satisfaction to a successful marriage relationship, and were concerned that the couples they counselled be sexually faithful. One counselor described his own sexually satisfying marriage of decades, colourfully stating, ‘I never forget to remind [counselees] that as long as they are together they must not sit still like furniture, but they must remember what is to be done in the bedroom.’

### Relationship satisfaction

With the exception of three women, all participants in the life-course interviews reported that they were generally satisfied and happy in their relationships. Yet some participants who reported that they were satisfied in the relationship, particularly women, also reported serious relationship issues or that the relationship differed significantly from their ideal relationship. Relationship problems included partners not helping with housework or abusing alcohol, lack of progress towards marriage, household poverty, suspected or known infidelity, lack of sexual satisfaction and physical or sexual violence.

The life-course interviews yielded particularly rich data on relationship satisfaction over time. In many cases, a participant’s stated level of relationship satisfaction was belied by events which transpired over the course of the research, such as infidelity or the ending of a relationship. As an example, two women (both partnered, one in 20s, one in 30s) chose to separate from their partners not long after they reported that they were ‘99% satisfied’ in their relationships. One of these women (20s) paradoxically stated in the same interview that her partner’s drinking was ‘not a serious problem,’ and that if he didn’t stop drinking she would end the relationship, a threat which she carried out.

In contrast, during life-course interviews, three women expressed very low satisfaction with relationships that had been characterised by physical violence and abuse and lack of emotional and financial support. One woman (30s, partnered) said she felt she had ‘lost her dignity’ through her on-again, off-again relationship of some years, which had involved multiple infidelities and serious physical violence. She stated that love, trust, and ‘believing in each other’ were essential to a good relationship, but when asked by the interviewer if she had these things in her relationship, she replied,

It’s not there. Even if [my partner] is just going to the toilet I won’t believe that he actually went there. I no longer trust him and that affects our love as now I don’t believe in our love and I have the fear that at any time things will be over and for real … I’m hurt and I just want to start a new life, just stay away from him.

Male participants in the life-course interviews universally reported that they were happy in their relationships, although 6 of 14 men also cheated on their partners during the research period. Two of these men who had themselves been unfaithful (both partnered, 20s) reported great unhappiness when their girlfriends cheated or broke off the relationship during the research.

FGD participants expressed that they thought most people in Eswatini were not very satisfied with their relationships, due especially to men’s infidelity. One man commented,

People’s salaries, about 25% of them are spent on people who we’re in love with outside our [primary] relationships. That means our wives are not satisfied when it comes to money, or even sexually.

In another FGD, a man was asked if people were satisfied in their relationships and replied,

Not really! Some people will confess that they are satisfied yet they are not. Some people will have everything in a relationship but you find that they are busy running after girls and if you ask that person what is he doing, he cannot answer you.

With a few exceptions, men and women who were married or in more long-standing and committed relationships expressed greater relationship satisfaction. Men and women who had received marriage counselling (accounting for the majority of married women and men in this study) reported being particularly satisfied in their marriages, and said they felt counselling had had a positive impact on the quality of their relationships or even saved their relationships when they were faced with challenges. Many counselees also reported being asked for relationship advice by friends or neighbours, suggesting that others recognised their relationships as worthy of emulation. Counselors provided many examples of couples they felt they had helped to have stronger relationships, deal with serious relationship issues (such as violence, abuse, infidelity, and HIV infection), and avoid separation or divorce. One female counselee (30s) reported,

I wanted to have a healthy and happy marriage, but at the beginning of our marriage we had a lot of problems and I didn’t think that I would ever enjoy being married. But now things have changed. We are at peace, we love each other, and there is trust.

All participants in the life-course interviews were asked at the conclusion of the study whether they would be interested in meeting with a trained counselor, and were given information on counselling services if they desired it. FGD participants were also given this information. Among life-course interview participants, 11 of 14 women and 7 of 14 men reported that they were interested in seeking counselling immediately or in the future, and one man and one woman did seek counselling during the study period. Participants expressed a particular desire to learn to communicate better, such as the woman (20s, partnered) who said,

I would like to know how can we work on communicating better and resolving our problems without fighting … We have to be faithful and learn to communicate as most relationships have this problem and then people they start having these extra relationships because they are having problems with their partners which they can’t work out.

Although the study was not intended to have a therapeutic effect, some participants in the life-course interviews and FGDs felt that they had been helped by having the opportunity to ‘cough out some issues,’ ‘pour out [their] hearts,’ and ‘share some secrets.’ Six participants in the life-course interviews (all women) expressed a desire for another such opportunity to sit and discuss their relationships. One woman (30s, partnered) said that participating in the study ‘helped me a lot and I wish you could arrange more workshops, even if you don’t offer us anything but just to sit and talk about how we can stay faithful and honest in our relationships.’ Several male and female FGD participants asked when another such discussion would be held, as they had found it helpful and wanted to participate again. One participant in the life-course interviews (20s) credited her participation in the study with helping her and her partner to decide to end their relationships with outside partners and commit to each other, with her partner beginning traditional marriage proceedings during the course of the study.

## Discussion

This qualitative study revealed seven characteristics of good relationships to be most salient among Swazi adults: love, respect, trust, honesty, good communication, sexual satisfaction, and sexual faithfulness. To our knowledge, this study is the first to describe dimensions of relationship satisfaction and relationship quality among Swazis, and among the first in sub-Saharan Africa. Comparison of our findings to research from the United States and Europe reveals significant similarities, which suggests that many aspects of couple relationships may be comparable across cultures. The seven key relationship characteristics identified by this study correspond quite closely to five dimensions of relationship quality which emerged from a review of the close relationships literature (Lawrence et al., 2008), as shown in Table 3. While we find these similarities striking, we also recognise the need for further research regarding how the meaning of these constructs might differ between cultures. We also note that participants in this study often struggled to define constructs such as love or trust, and that boundaries between constructs were often indistinct. This suggests that there are intersections between relationship constructs which might further complicate efforts to define and measure them in different cultural contexts.

**Table 3.** Comparison of dimensions of relationship quality.

Characteristics of a good relationship (present study)	Dimensions of relationship quality (Lawrence et al., 2008)
(1) trust and honesty	(1) trust, closeness, and emotional intimacy
(2) love (*understood in terms of financial, practical, and emotional support*)	(2) inter-partner support (*understood as emotional or tangible support when a partner has a problem or is ‘down’*)
(3) sexual faithfulness and sexual satisfaction	(3) quality of the sexual relationship
(4) respect	(4) respect, power, and control
(5) communication	(5) communication and conflict management

Love and respect were the two most prominent dimensions of couple relationships in this study. Amato (2007) writes that despite love being the primary reason that men and women enter relationships and marry, there is a lack of serious research about the subject. With some notable exceptions (Cole & Thomas, 2009; Hunter, 2010; Lesch & Adams, 2016; Mojola, 2014; Parikh, 2016; Smith, 2001; Sølbeck, 2010), this deficit of research is even greater with respect to Africa (Ruark et al., 2017). Whereas historians have argued that romantic love has long been part of African cultures, romantic love as a basis for marriage or other long-term relationships is a more modern phenomenon (Vaughan, 2010). Based on rich ethnography conducted in Kwa-Zulu Natal, South Africa (a region geographically and culturally contiguous to Eswatini), Hunter (2010) describes a modern amalgamation of provider love and romantic love. Provider love encompasses exchange of money and other such practical expressions, whereas romantic love is characterised by expressions of passion and companionate partnerships.

Some HIV prevention research has given attention to the importance of love. A study of Ugandan couples’ adherence to HIV pre-exposure prophylaxis (PrEP) found that when faced with HIV sero-discordance, many couples were strongly motivated to continue the relationship because they loved each other (Ware et al., 2012). In a study of relationship expectations and HIV risk among young women in inner-city Johannesburg, South Africa, young women expressed that romantic relationships should be based on love, respect, mutual support, and mutual sexual fidelity, although their relationships were often characterised by harsher realities such as violence and infidelity (Pettifor, MacPhail, Anderson, & Maman, 2012).

Respect emerged as a major theme of our study, but has not been a major focus of couples research globally (S. S. Hendrick & Hendrick, 2006). According to analysis by Lawrence and colleagues (2008), psychologists have primarily associated respect in couple relationships with the constructs of power and control. Research has demonstrated that power imbalances in intimate relationships are associated with lower relationship satisfaction (Bentley, Galliher, & Ferguson, 2007; Whisman & Jacobson, 1990) and greater likelihood of relationship dissolution (DeMaris, 2007). Swazi men and women did describe elements of power and control, yet also frequently discussed respect not in terms of control but in terms of deference and honour. Dew and Wilcox (2013) may come closer to a Swazi understanding of respect when they recognise displays of respect as an element of generosity, along with ‘small acts of kindness,’ displays of affection, and forgiveness.

Hunter defines respect (*hlonipha*) in KwaZulu-Natal, South Africa as an ‘embodied sense of acts of deference,’ including those that traditionally positioned wives as subservient to husbands (2010, p. 10). In this study, the emphasis that women gave to receiving respect from men (an emphasis that was not present when men discussed how they should treat women) may be an indication of women experiencing a deficit of respect from partners, and their dissatisfaction with this reality. Hunter (2010) also describes how men have traditionally earned respect through marriage and providing materially for one or more wives. Thus respect becomes entwined with ‘expressions of love enacted through cooperation and mutual assistance,’ which Hunter terms provider love (2010, p. 16). More research is needed to explore the ways that couples may define and value respect in various cultures, and the impact on relationship quality and satisfaction.

Whereas the interview guides used in this study did not introduce HIV as a topic of discussion, the frequency with which participants mentioned HIV indicated that the epidemic provided an ever-present backdrop to their sexual partnerships. Participants’ concerns about honesty and trust in their relationships were closed linked to concerns regarding whether a partner might be bringing HIV into the relationship. Similarly, a recent study found that some South African women and men reported loving their partners but not completely trusting them, and did not consider lack of trust as a reason to end a relationship (Parker et al., 2014). Very little research has explored the degree to which subjective perceptions of trust in a relationship are correlated to HIV risk in a relationship, although a qualitative study in Uganda found that HIV infection was correlated with men’s and women’s perceptions of trust and sexual faithfulness within their relationships (Higgins et al., 2014). Participants in this study who reported low levels of trust and honesty also demonstrated anxiety about the future of their relationships, as well as a reluctance to emotionally engage in the relationship.

The importance that Swazi women and men ascribed to communication is consistent with the view of U.S. experts that good communication is a vital relationship skill (Hawkins, Carroll, Doherty, & Willoughby, 2004). In African contexts, lack of communication has been found to impede couples’ ability to deal with an HIV diagnosis (Medley, Kennedy, Lunyolo, & Sweat, 2009) whereas when couples build their communication skills they are more equipped to resolve relationship challenges, including those related to HIV (Conroy, McGrath, et al., 2016). A recent intervention aimed at reducing HIV risk among South African couples through strengthening communication and other relationship skills succeeded in increasing gender equity and decreasing IPV (Minnis et al., 2015). Participants in this research expressed that communication was the aspect of couple relationships for which they most desired support and education, and counselors also emphasised building communication skills.

A large literature on sexual satisfaction among U.S. populations shows an unequivocal link between sexual satisfaction and overall relationship satisfaction (Byers, 2005; Schenk, Pfrang, & Rausche, 1983; Schwartz & Young, 2009), and additionally between sexual satisfaction and overall physical and mental well-being (R. M. Anderson, 2013). Participants in our study insisted that lack of sexual satisfaction was a major reason people engaged in multiple and concurrent sexual partnerships. These findings are consistent with research from Kenya (Kwena, Mwanzo, Bukusi, Achiro, & Shisanya, 2014), Malawi (Tawfik & Watkins, 2007), Tanzania (Cox et al., 2014), and Zimbabwe (Mugweni, Pearson, & Omar, 2015) which has found that women and men who engage in multiple and concurrent sexual partnerships, thus placing themselves at greater risk of HIV, often are motivated by a lack of relationship satisfaction and particularly sexual satisfaction in their primary relationships. Despite this research, we observe that HIV prevention interventions often fail to recognise lack of sexual satisfaction as a component of HIV risk. While male and female participants in this study repeatedly expressed the desire for partners who were sexually faithful, participants in the life-course interviews ranked sexual faithfulness relatively low in the ranking exercise (11th of 15 items among women, 8th of 15 items among men). This may reflect their realisation and resignation that their relationships often did not involve sexual exclusivity.

Women in our study generally reported lower relationship satisfaction than men. Men may have simply been less willing to talk about problems and lack of satisfaction in their relationships. Based on our data and knowledge of Swazi culture, we theorise that women in Eswatini do feel significantly less satisfaction in their relationships, due both to unequal power dynamics and because they have less ability or inclination to leave unsatisfactory relationships, compared to men. If such a relationship satisfaction gap does exist, this would contrast with developed countries, where women and men have been found to have comparable levels of marital satisfaction (Jackson, Miller, Oka, & Henry, 2014). Such a cross-cultural difference might signify that Swazi women experience greater gender inequities and power imbalances within relationships than do women in other contexts. Women’s lack of power in intimate sexual partnerships is a health issue, and has been linked to lower relationship quality in South Africa (Conroy, McGrath, et al., 2016) and increased risk of sexual violence in Uganda (Conroy, Tsai, et al., 2016) and South Africa (Minnis et al., 2015).

This study revealed multiple instances in which participants in the life-course interviews seemed to be unable to recognise or admit aspects of a partner or partnership which to the study team seemed troubling or negative. In some cases, participants depicted their relationships as being held together with strong bonds of love, respect, and trust, only to report a few months later that a partner had deceived or abused them, engaged in a concurrent sexual partnership, or that the relationship had otherwise come under strain or dissolved. The Swazi saying ‘*kugigwa lutsandvo*’ (literally ‘bound by love’), much like the English phrase ‘love is blind,’ is used to describe a situation in which a person in love loses the ability to accurately discern reality. Far from being a phenomenon unique to this research context, this tendency may be seen as a form of sentiment override (Hawkins, Carrère, & Gottman, 2002). In our study, women ‘blinded by love’ seemed particularly apt to, as Amato describes, ‘attribute their spouses’ bad behaviour to external and uncontrollable causes rather than to internal and controllable causes’ (Amato, 2007, p. 307). Being ‘blinded’ or ‘bound’ by love may be one factor which leads women to ignore risks to their health and well-being and remain in poor quality or abusive relationships.

Whereas some participants in the life-course interviews (particularly women) seem to have been ‘blinded by love’, others may have presented idealised accounts of their relationship for reasons of social desirability. For example, some men expressed ideals of honesty and sexual faithfulness which they admitted they did not attain in their relationships. A similar disjuncture between personal relationship ideals and actual experience has been noted among young Malawians, where this ‘distance’ was linked to poorer relationship outcomes in terms of emotional support, communication, relationship stability, and perceived risk of HIV infection (Frye & Trinitapoli, 2015). Another possible explanation for discrepant accounts of relationship quality and satisfaction is that in a context of low relational expectations, individuals are relatively satisfied with poor-quality relationships. Low correlation between relationship quality and relationship satisfaction has been noted by other researchers (Lawrence et al., 2011).

We also note discrepancies between data sources. The predominantly negative views expressed by FGD participants about the state of relationships in Eswatini diverged strikingly from the more positive accounts given by individual participants during in-depth interviews. One explanation is that interview participants may have been more likely to be affected not only by sentiment override but also by social desirability bias, both of which would have caused them to present an idealised version of their relationships. In contrast, the prevailing negative tone of FGDs may have caused participants in the FGDs to dwell on negative aspects of their own relationships rather than more positive aspects. Both possibilities speak to the fact that participants do not present their stories in a vacuum, but rather co-construct a version of reality through interaction with other participants in the research process.

### Limitations and directions for future research

This study draws on multiple sources of data, including lengthy, in-depth life-course interviews with participants with whom the interviewers built trust and rapport over time. The ability to triangulate between different sources of data was a strength of this study, and allows us to examine various and sometimes contradictory claims regarding relationships in Eswatini and produce a more nuanced depiction than could be obtained from one data source alone. Nevertheless, various biases are likely present, including social desirability and recall bias, as participants may have chosen to both remember and represent their personal histories in overly sanguine terms. Men, who reported greater relationship satisfaction, may have been particularly likely to conceal relationship difficulties or areas of relationship dissatisfaction. Counselees (male and female) were generally more willing to talk about difficulties in their relationships, perhaps because the interviewer already knew they had sought counselling, and because their counselling encounters had given them experience in talking about their relationships. The stories and opinions represented here are unique to individuals and cannot be assumed to be representative of Swazis as a whole, and this is likely particularly true of counselors and counselees, who were highly unusual in their cultural context (by virtue of either offering or seeking marriage counselling). These caveats aside, the similarities in experiences and beliefs among participants suggested many shared cultural norms and values regarding relationship quality and satisfaction.

This study was designed to provide thick description of sexual relationships in Eswatini and contribute to development of theory about dimensions of relationship quality and satisfaction among Swazis. We believe that these findings may also have relevance for other African populations. Indeed, comparison of our findings to previous research on couple relationships in Africa reveals many common threads, suggesting that Swazi couples may have many similar relationship dynamics to couples elsewhere on the continent. Our survey of the global literature suggested that people all over the world may define a ‘good relationship’ in quite similar ways, yet also highlighted distinctives, such as that respect may be a particularly salient aspect of relationships in Eswatini.

We suggest several avenues of further research. First, these findings might be used to develop or adapt psychosocial measures of relationship quality and satisfaction for use in Eswatini and similar sociocultural contexts. Such measurement could reveal much about relationship quality and satisfaction in Eswatini (including differences by gender and marital status) and contribute to the study of cross-cultural similarity and difference, including cross-cultural studies within Africa. Furthermore, the validation of these measures could pave the way for studies of how relationship quality and satisfaction are linked to mental and physical health, including HIV risk, in Eswatini and elsewhere in Africa. Finally, we hope that a deeper understanding of couple functioning in an African population will contribute to interventions which seek to strengthen couple relationships in Africa, recognising that models of marriage and relationship education from high-resource Western contexts may need significant modification to be effective in other regions of the world.

## Conclusion

This research finds significant common ground between Swazis’ understandings of a good relationship and the characteristics of relationship quality identified by research elsewhere in the world, suggesting that expectations for what constitutes a good relationship may be comparable across cultures and similarly rooted in the construct of love. A second key finding of this research is that Swazis reported a significant disjuncture between their relationship ideals and relational realities, particularly in the areas of trust, honesty, and sexual faithfulness. Although relationship quality and relationship satisfaction have been rarely explored in Eswatini or other parts of Africa, research from other cultures suggests that these aspects of human experience are critically related to individual mental, emotional, and physical health. The interconnections between the functioning of intimate sexual relationships and overall health may be particularly consequential in Eswatini, where women and men are at high risk of HIV infection. Further research is needed to establish how couple relationships may be strengthened in Eswatini through culturally-grounded, sustainable approaches.

## Footnotes

1 Although there is a lack of consensus among researchers regarding definitions of relationship satisfaction and relationship quality, relationship satisfaction is generally understood as a one-dimensional construct referring to a general feeling of happiness in a relationship, whereas relationship quality encompasses a number of different dimensions (Lawrence, Brock, Barry, Langer, & Bunde, 2008). Relationship satisfaction is an inherently subjective construct, and some researchers have argued that relationship quality should be assessed according to more objective definitions of a ‘good relationship’ and that satisfaction should not be used as a proxy for quality (Knapp & Lott, 2010). Relationship satisfaction and quality are not necessarily correlated, as an individual may feel high satisfaction with a low-quality relationship, or vice-versa, depending in part on his or her expectations for the relationship. Thus we choose to distinguish between relationship satisfaction and quality in this article, and give greater emphasis to relationship quality as a more multi-dimensional and less subjective construct with which to examine couple relationships.

2 Calculations made using data from the 2006/2007 *Swaziland Demographic and Health Survey*, available at www.measuredhs.com.

## References

[CIT0001] Amato P. R. (2007). Transformative processes in marriage: Some thoughts from a sociologist. *Journal of Marriage and Family*, 69, 305–309. doi: 10.1111/j.1741-3737.2007.00365.x

[CIT0002] Anderson R. M. (2013). Positive sexuality and its impact on overall well-being. *Bundesgesundheitsblatt, Gesundheitsforschung, Gesundheitsschutz*, 56(2), 208–214. doi: 10.1007/s00103-012-1607-z 23361205

[CIT0003] Belus J. M., Kline T., Carney T., Myers B., & Wechsberg W. M. (2018). Measuring relationship functioning in South African couples: A strategy for improving HIV prevention efforts. *Sexual and Relationship Therapy*, 10(7). doi: 10.1080/14681994.2017.1419559 PMC7388832 32728347

[CIT0004] Bentley C. G., Galliher R. V., & Ferguson T. J. (2007). Associations among aspects of interpersonal power and relationship functioning in adolescent romantic couples. *Sex Roles*, 57(7–8), 483–495. doi: 10.1007/s11199-007-9280-7 18776943 PMC2529377

[CIT0005] Bicego G. T., Nkambule R., Peterson I., Reed J., Donnell D., Ginindza H., … Justman J. (2013). Recent patterns in population-based HIV prevalence in Swaziland. *Plos One*, 8(10), e77101. doi: 10.1371/journal.pone.0077101 24143205 PMC3797108

[CIT0006] Blanc A. K. (2001). The effect of power in sexual relationships on sexual and reproductive health: An examination of the evidence. *Studies in Family Planning*, 32(3), 189–213. doi: 10.1111/j.1728-4465.2001.00189.x 11677692

[CIT0007] Bongaarts J. (2007). Late marriage and the HIV epidemic in sub-Saharan Africa. *Population Studies*, 61(1), 73–83. doi: 10.1080/00324720601048343 17365874

[CIT0008] Brenner M. (1985). An introduction to content analysis. In *The research interview: Uses and approaches* (pp. 238–267). New York, NY: Academic Press.

[CIT0009] Brown S. L., Bulanda J. R., & Lee G. R. (2005). The significance of nonmarital cohabitation: Marital status and mental health benefits among middle-aged and older adults. *The Journals of Gerontology Series B: Psychological Sciences and Social Sciences*, 60(1), S21–S29. doi: 10.1093/geronb/60.1.S21 15643043

[CIT0010] Butler S. (2008). Mall intercept survey. In *Encyclopedia of survey research methods* (pp. 448–449). Thousand Oaks, CA: Sage.

[CIT0011] Byers E. S. (2005). Relationship satisfaction and sexual satisfaction: A longitudinal study of individuals in long-term relationships. *Journal of Sex Research*, 42(2), 113–118. doi: 10.1080/00224490509552264 16123841

[CIT0012] Carr D., Freedman V. A., Cornman J. C., & Schwarz N. (2014). Happy marriage, happy life? Marital quality and subjective well-being in later life. *Journal of Marriage and Family*, 76(5), 930–948. doi: 10.1111/jomf.12133 25221351 PMC4158846

[CIT0013] Central Statistics Office (CSO) & Macro International Inc (2008). *Swaziland Demographic and Health Survey 2006-07*. Mbabane: Author.

[CIT0014] Clark S. (2004). Early marriage and HIV risks in Sub-Saharan Africa. *Studies in Family Planning*, 35(3), 149–160. 15511059 10.1111/j.1728-4465.2004.00019.x

[CIT0015] Cole J., & Thomas L. M. (Eds.). (2009). *Love in Africa*. Chicago, IL: University of Chicago Press.

[CIT0016] Conroy A., Leddy A., Johnson M., Ngubane T., van Rooyen H., & Darbes L. (2017). “I told her this is your life”: Relationship dynamics, partner support and adherence to antiretroviral therapy among South African couples. *Culture, Health & Sexuality*, 19(11), 1239–1253. doi: 10.1080/13691058.2017.1309460 PMC5626574 28398134

[CIT0017] Conroy A. A. (2014). “It means there is doubt in the house”: Perceptions and experiences of HIV testing in rural Malawi. *Culture, Health & Sexuality*, 16(4), 397–411. doi: 10.1080/13691058.2014.883645 PMC3991518 24580127

[CIT0018] Conroy A. A. (2015). The influence of relationship power dynamics on HIV testing in rural Malawi. *The Journal of Sex Research*, 52(3), 347–359. doi: 10.1080/00224499.2014.883590 24670263 PMC4177026

[CIT0019] Conroy A. A., McGrath N., van Rooyen H., Hosegood V., Johnson M. O., Fritz K., … Darbes L. A. (2016). Power and the association with relationship quality in South African couples: Implications for HIV/AIDS interventions. *Social Science & Medicine*, 153, 1–11. doi: 10.1016/j.socscimed.2016.01.035 26859436 PMC4788545

[CIT0020] Conroy A. A., McKenna S. A., & Ruark A. (2019). Couple interdependence impacts alcohol use and adherence to antiretroviral therapy in Malawi. *AIDS and Behavior*, 23(1), 201–210. doi: 10.1007/s10461-018-2275-2 30218319 PMC7232967

[CIT0021] Conroy A. A., Tsai A. C., Clark G. M., Boum Y., Hatcher A. M., Kawuma A., … Weiser S. D. (2016). Relationship power and sexual violence among HIV-positive women in rural Uganda. *AIDS and Behavior*, 20(9), 2045–2053. doi: 10.1007/s10461-016-1385-y 27052844 PMC4996683

[CIT0022] Cox C. M., Babalola S., Kennedy C., Mbwambo J. K., Likindikoki S., & Kerrigan D. (2014). Determinants of concurrent sexual partnerships within stable relationships: A qualitative study in Tanzania. *BMJ Open*, 4, e003680. doi: 10.1136/bmjopen-2013-003680 PMC3918978 24508848

[CIT0023] Creswell J. A. (2013). Five qualitative approaches to inquiry. In *Qualitative inquiry and research design: Choosing among five approaches* (pp. 53–84). Thousand Oaks, CA: Sage.

[CIT0024] Darbes L. A., van Rooyen H., Hosegood V., Ngubane T., Johnson M. O., Fritz K., & McGrath N. (2014). Uthando Lwethu (“our love”): A protocol for a couples-based intervention to increase testing for HIV: A randomized controlled trial in rural KwaZulu-Natal, South Africa. *Trials*, 15(64). doi: 10.1186/1745-6215-15-64 PMC3936910 24552199

[CIT0025] DeMaris A. (2007). The role of relationship inequity in marital disruption. *Journal of Social and Personal Relationships*, 24(2), 177–195. doi: 10.1177/0265407507075409

[CIT0026] Dew J., & Wilcox W. B. (2013). Generosity and the maintenance of marital quality. *Journal of Marriage and Family*, 75(5), 1218–1228. doi: 10.1111/jomf.12066

[CIT0027] Drefahl S. (2012). Do the married really live longer? The role of cohabitation and socioeconomic status. *Journal of Marriage and Family*, 74(3), 462–475. doi: 10.1111/j.1741-3737.2012.00968.x

[CIT0028] Dunkle K. L., Jewkes R. K., Brown H. C., Gray G. E., McIntryre J. A., & Harlow S. D. (2004). Gender-based violence, relationship power, and risk of HIV infection in women attending antenatal clinics in South Africa. *The Lancet*, 363(9419), 1415–1421. doi: 10.1016/S0140-6736(04)16098-4 15121402

[CIT0029] Frye M., & Trinitapoli J. (2015). Ideals as anchors for relationship experiences. *American Sociological Review*, 80(3), 496–525. doi: 10.1177/0003122415581333 27110031 PMC4838189

[CIT0030] Gevers A., Jewkes R., & Mathews C. (2013). What do young people think makes their relationships good? Factors associated with assessments of dating relationships in South Africa. *Culture, Health & Sexuality*, 15(9), 1011–1025. doi: 10.1080/13691058.2013.803295 PMC3795933 23805889

[CIT0031] Giele J. Z., & Elder G. H. Jr (1998). Life course research: Development of a field. In *Methods of life-course research: Qualitative and quantitative approaches* (pp. 5–27). Thousand Oaks, CA: Sage.

[CIT0032] Hatcher A. M., Tsai A. C., Kumbakumba E., Dworkin S. L., Hunt P. W., Martin J. N., … Weiser S. D. (2012). Sexual relationship power and depression among HIV-infected women in rural Uganda. *Plos One*, 7(12), e49821. doi: 10.1371/journal.pone.0049821 23300519 PMC3530575

[CIT0033] Hawkins A. J., Carroll J. S., Doherty W. J., & Willoughby B. (2004). A comprehensive framework for marriage education. *Family Relations*, 53(5), 547–558.

[CIT0034] Hawkins M. W., Carrère S., & Gottman J. M. (2002). Marital sentiment override: Does it influence couples’ perceptions? *Journal of Marriage and Family*, 64(1), 193–201.

[CIT0035] Hendrick S. S., & Hendrick C. (2006). Measuring respect in close relationships. *Journal of Social and Personal Relationships*, 23(6), 881–899. doi: 10.1177/0265407506070471

[CIT0036] Higgins J. A., Mathur S., Eckel E., Kelly L., Nakyanjo N., Sekamwa R., … Santelli J. S. (2014). Importance of relationship context in HIV transmission: Results from a qualitative case-control study in Rakai, Uganda. *American Journal of Public Health*, 104(4), 612–620. doi: 10.2105/AJPH.2013.301670 24524490 PMC4025686

[CIT0037] Hsieh H.-F., & Shannon S. E. (2005). Three approaches to qualitative content analysis. *Qualitative Health Research*, 15(9), 1277–1288. doi: 10.1177/1049732305276687 16204405

[CIT0038] Hunter M. (2010). *Love in the time of AIDS: Inequality, gender, and rights in South Africa*. Bloomington: Indiana University Press.

[CIT0039] Jackson J. B., Miller R. B., Oka M., & Henry R. G. (2014). Gender differences in marital satisfaction: A meta-analysis. *Journal of Marriage and Family*, 76(1), 105–129. doi: 10.1111/jomf.12077

[CIT0040] Jewkes R. K., Dunkle K. L., Nduna M., & Shai N. (2010). Intimate partner violence, relationship power inequity, and incidence of HIV infection in young women in South Africa: A cohort study. *The Lancet*, 376(9734), 41–48. doi: 10.1016/S0140-6736(10)60548-X 20557928

[CIT0041] Justman J., Reed J. B., Bicego G., Donnell D., Li K., Bock N., … Nkambule R. (2017). Swaziland HIV incidence measurement survey (SHIMS): A prospective national cohort study. *The Lancet HIV*, 4(2), e83–e92. doi: 10.1016/S2352-3018(16)30190-4 27863998 PMC5291824

[CIT0042] Knapp S. J., & Lott B. (2010). Forming the central framework for a science of marital quality: An interpretive alternative to marital satisfaction as a proxy for marital quality. *Journal of Family Theory & Review*, 2(4), 316–333. doi: 10.1111/j.1756-2589.2010.00064.x

[CIT0043] Koskinen S., Joutsenniemi K., Martelin T., & Martikainen P. (2007). Mortality differences according to living arrangements. *International Journal of Epidemiology*, 36(6), 1255–1264. doi: 10.1093/ije/dym212 17971389

[CIT0044] Kwena Z. A., Mwanzo I. J., Bukusi E. A., Achiro L. F., & Shisanya C. A. (2014). Across-sectional survey of prevalence and correlates of couple sexual concurrency among married couples in fishing communities along Lake Victoria in Kisumu, Kenya. *Sexually Transmitted Infections*, 90(2), 139–144. doi: 10.1136/sextrans-2013-051168 24154655 PMC5608652

[CIT0045] Lawrence E., Barry R. A., Brock R. L., Bunde M., Langer A., Ro E., … Dzankovic S. (2011). The relationship quality interview: Evidence of reliability, convergent and divergent validity, and incremental utility. *Psychological Assessment*, 23(1), 44–63. doi: 10.1037/a0021096 21280953

[CIT0046] Lawrence E., Brock R. L., Barry R. A., Langer A., & Bunde M. (2008). Assessing relationship quality: Development of an interview and implications for couple assessment and intervention. In *Psychology of relationships* (pp. 173–189). Hauppauge: Nova Science.

[CIT0047] Lesch E., & Adams A. R. (2016). Sexual intimacy constructions of heterosexual couples living in a low-income, “colored,” farmworker community in South Africa. *The Journal of Sex Research*, 53(9), 1082–1095. doi: 10.1080/00224499.2016.1144170 26986557

[CIT0048] Lincoln Y. S., & Guba E. G. (1985). *Naturalistic inquiry* (1st ed.). Thousand Oaks, CA: Sage.

[CIT0049] Maman S., Mbwambo J., Hogan N. M., Kilonzo G. P., & Sweat M. (2001). Women’s barriers to HIV-1 testing and disclosure: Challenges for HIV-1 voluntary counselling and testing. *AIDS Care*, 13(5), 595–603. doi: 10.1080/09540120120063223 11571006

[CIT0050] Marcussen K. (2005). Explaining differences in mental health between married and cohabiting individuals. *Social Psychology Quarterly*, 68(3), 239–257. doi: 10.1177/019027250506800304

[CIT0051] Mastekaasa A. (1994). Marital status, distress, and well-being: An international comparison. *Journal of Comparative Family Studies*, 25(2), 183–205.

[CIT0052] Medley A. M., Kennedy C. E., Lunyolo S., & Sweat M. D. (2009). Disclosure outcomes, coping strategies, and life changes among women living with HIV in Uganda. *Qualitative Health Research*, 19(12), 1744–1754. doi: 10.1177/1049732309353417 19949223

[CIT0053] Minnis A., Doherty I., Kline T., Zule W., Myers B., Carney T., & Wechsberg W. (2015). Relationship power, communication, and violence among couples: Results of a cluster-randomized HIV prevention study in a South African township. *International Journal of Women’s Health*, 7, 517–525. doi: 10.2147/IJWH.S77398 PMC4435250 25999767

[CIT0054] Mojola S. A. (2014). *Love, money, and HIV: Becoming a modern African woman in the age of AIDS*. Berkeley: University of California Press.

[CIT0055] Mugweni E., Pearson S., & Omar M. (2015). Concurrent sexual partnerships among married Zimbabweans – implications for HIV prevention. *International Journal of Women’s Health*, 7, 819–832. doi: 10.2147/IJWH.S88884 PMC4599069 26491372

[CIT0056] National Emergency Response Council on HIV and AIDS (NERCHA) (2009). *Swaziland HIV prevention response and modes of transmission analysis*. Mbabane: NERCHA.

[CIT0057] Ndlangamandla E. (2007). *Swaziland human development report: HIV and AIDS and culture*. Swaziland: United Nations Development Programme (UNDP Retrieved from http://hdr.undp.org/sites/default/files/swaziland_nhdr_2008.pdf

[CIT0058] Parikh S. A. (2016). *Regulating romance: Youth love letters, moral anxiety, and intervention in Uganda’s time of AIDS*. Nashville, TN: Vanderbilt University Press.

[CIT0059] Parker L., Pettifor A., Maman S., Sibeko J., & MacPhail C. (2014). Concerns about partner infidelity are a barrier to adoption of HIV-prevention strategies among young South African couples. *Culture, Health & Sexuality*, 16(7), 792–805. doi: 10.1080/13691058.2014.905707 PMC4832568 24816215

[CIT0060] Perelli-Harris B., & Styrc M. (2018). Mental well-being differences in cohabitation and marriage: The role of childhood selection. *Journal of Marriage and Family*, 80(1), 239–255. doi: 10.1111/jomf.12431 29456265 PMC5811838

[CIT0061] Pettifor A., MacPhail C., Anderson A. D., & Maman S. (2012). “If I buy the Kelloggs then he should [buy] the milk”: Young women’s perspectives on relationship dynamics, gender power and HIV risk in Johannesburg, South Africa. *Culture, Health & Sexuality*, 14(5), 477–490. doi: 10.1080/13691058.2012.667575 PMC3570821 22449022

[CIT0062] Proulx C. M., Helms H. M., & Buehler C. (2007). Marital quality and personal well-being: A meta-analysis. *Journal of Marriage and Family*, 69(3), 576–593. doi: 10.1111/j.1741-3737.2007.00393.x

[CIT0063] Ren X. S. (1997). Marital status and quality of relationships: The impact on health perception. *Social Science & Medicine*, 44(2), 241–249. doi: 10.1016/S0277-9536(96)00158-X 9015876

[CIT0064] Ruark A., Dlamini L., Mazibuko N., Green E. C., Kennedy C., Nunn A., … Surkan P. J. (2014). Love, lust and the emotional context of multiple and concurrent sexual partnerships among young Swazi adults. *African Journal of AIDS Research*, 13(2), 133–143. 25174630 10.2989/16085906.2014.927781 PMC4201849

[CIT0065] Ruark A., Kennedy C.E., Mazibuko N., Dlamini L., Nunn A., Green E.C., & Surkan P. J. (2016). From first love to marriage and maturity: A life-course perspective on HIV risk among young Swazi adults. *Culture, Health & Sexuality*, 18(7), 812–825. doi: 10.1080/13691058.2015.1134811 PMC4877271 26901064

[CIT0066] Ruark A., Stern E., Dlamini-Simelane T., & Kakuze M. F. (2017). Love matters: Exploring conceptions of love in Rwanda and Swaziland and relationship to HIV and intimate partner violence. *African Journal of AIDS Research*, 16(4), 271–282. doi: 10.2989/16085906.2017.1343740 29132284 PMC5907914

[CIT0067] Schenk J., Pfrang H., & Rausche A. (1983). Personality traits versus the quality of the marital relationship as the determinant of marital sexuality. *Archives of Sexual Behavior*, 12(1), 31–42. doi: 10.1007/BF01542114 6838353

[CIT0068] Schwartz P., & Young L. (2009). Sexual satisfaction in committed relationships. *Sexuality Research and Social Policy*, 6(1), 1–17. doi: 10.1525/srsp.2009.6.1.1

[CIT0069] Shisana O., Risher K., Celentano D. D., Zungu N., Rehle T., Ngcaweni B., & Evans M. G. (2016). Does marital status matter in an HIV hyperendemic country? Findings from the 2012 South African National HIV prevalence, incidence and behaviour survey. *AIDS Care*, 28(2), 234–241. doi: 10.1080/09540121.2015.1080790 26551532 PMC5146982

[CIT0070] Siedner M. J., Tsai A. C., Dworkin S., Mukiibi N. F. B., Emenyonu N. I., Hunt P. W., … Weiser S. D. (2012). Sexual relationship power and malnutrition among HIV-positive women in rural Uganda. *AIDS and Behavior*, 16(6), 1542–1548. doi: 10.1007/s10461-012-0162-9 22382629 PMC3439197

[CIT0071] Smith D. J. (2001). Romance, parenthood, and gender in a modern African society. *Ethnology*, 40(2), 129–151. doi: 10.2307/3773927

[CIT0072] Sølbeck D. E. (2010). “Love of the heart”: Romantic love among young mothers in Mali. *Culture, Health & Sexuality*, 12(4), 415–427. doi: 10.1080/13691051003586906 20169480

[CIT0073] Tabana H., Doherty T., Rubenson B., Jackson D., Ekström A. M., & Thorson A. (2013). “Testing together challenges the relationship”: Consequences of HIV testing as a couple in a high HIV prevalence setting in rural South Africa. *Plos One*, 8(6), e66390. doi: 10.1371/journal.pone.0066390.t001 23824067 PMC3688905

[CIT0074] Tawfik L., & Watkins S. C. (2007). Sex in Geneva, sex in Lilongwe, and sex in Balaka. *Social Science & Medicine*, 64(5), 1090–1101. doi: 10.1016/j.socscimed.2006.10.002 17123678

[CIT0075] Vaughan M. (2010). The history of romantic love in sub-Saharan Africa: Between interest and emotion. *Proceedings of the British Academy*, 167, 1–25.

[CIT0076] Ware N. C., Wyatt M. A., Haberer J. E., Baeten J. M., Kintu A., Psaros C., … Bangsberg D. R. (2012). What's love got to do with it? Explaining adherence to oral antiretroviral pre-exposure prophylaxis for HIV-serodiscordant couples. *JAIDS Journal of Acquired Immune Deficiency Syndromes*, 59(5), 463–468. doi: 10.1097/QAI.0b013e31824a060b 22267018 PMC3826169

[CIT0077] Whisman M. A., & Jacobson N. S. (1990). Power, marital satisfaction, and response to marital therapy. *Journal of Family Psychology*, 4(2), 202–212. doi: 10.1037/0893-3200.4.2.202

[CIT0078] Whitson S., & El-Sheikh M. (2003). Marital conflict and health. *Aggression and Violent Behavior*, 8(3), 283–312. doi: 10.1016/S1359-1789(01)00067-2

[CIT0079] Wiik K. A., Keizer R., & Lappegård T. (2012). Relationship quality in marital and cohabiting unions across Europe. *Journal of Marriage and Family*, 74(3), 389–398. doi: 10.1111/j.1741-3737.2012.00967.x

